# Toward Extreme Lossy Compression Data to Enable Higher Data Rates MX

**DOI:** 10.1063/4.0000936

**Published:** 2025-10-27

**Authors:** Jean Jakoncic, Herbert J Bernstein

**Affiliations:** 1 Brookhaven National Laboratory, National Synchrotron Light Source II, Upton, NY, USA; 2 Fresh Pond Institute, Cambridge, MA, USA

## Abstract

At last, a useful lossy compression method is available for MX crystallographic data. One could achieve a compression ratio as high as 3000 to 1, but with data loss so high that structural data can’t be interpreted faithfully. On the other hand, our l_bnl_compress lossy-but-not-(too)-lossy compression application allows one flexibility to control the amount of compression to apply. We have successfully applied lossy compression to three test data-sets, representing an array of samples and experiments performed at state- of-the art synchrotron beamlines. These were a Hen- Egg-White Lysozyme data set from a crystal collected at 7.5 keV for S_SAD phasing, a data set from Thermolysin with a complexed fragment molecule from a fragment-screening campaign, and a data set from activated Spheugomonas CBASS Casp5 at high resolution. We present results on the compressed data for these three test cases with compression ratio ranging from 100:1 to 2000:1. To test the quality of compression, all compressed data were decompressed, and then they underwent data reduction and processing workflows. Processed data were used to determine the corresponding 3D crystal structures and will be presented.

Our results strongly support the application of lossy compression with achieved ratio of 300 to 1 for permanent data archival. In other words, one can archive 1 PB of raw uncompressed* data into a single 4 TB thumb drive. This is a significant finding since it indicates that one can store data sets from 200,000 single crystals into a 4 TB thumb drive.

The high compression of 300 to 1 resulted in computed electron density maps with few measurable differences, when compared with maps from uncompressed* data.

*Uncompressed data: data generated by the X-ray pixel-array detectors (PAD) routinely in use at synchrotron facilities, with lossless compression applied, generally BSLZ4 (bit-shuffle LZ4 compression). The compressions stated are compression on top of the lossless compression provided with PAD detectors. On average, one gets 10 GB for a 1800-frames data set from a 10 megapixel detector.

Compression ratios stated in the abstract are extra-compression ratios, on top of the BS-LZ4 compressions, crystallographers are used to working with.

Raw data are data coming from the Data Collection Unit, prior to any compression applied. Approximately one 16-bit integer per pixel, or 36 GB for a 1800 frames data set from a 10 megapixel detector.

